# Calculating fertility and childhood mortality rates from survey data using the DHS.rates R package

**DOI:** 10.1371/journal.pone.0216403

**Published:** 2019-05-24

**Authors:** Mahmoud Elkasabi

**Affiliations:** ICF, The Demographic and Health Surveys Program, Rockville, Maryland, United States of America; Universidade Federal de Minas Gerais, BRAZIL

## Abstract

The Demographic and Health Surveys (DHS) are a major source for many demographic and health indicators in developing countries. Although these indicators are well defined in the literature, using survey data to calculate some of these indicators has never been an easy task for data users. This paper presents the DHS.rates software, a user-friendly R package developed to calculate fertility indicators, such as the total fertility rate, general fertility rate, and age-specific fertility rates, and childhood mortality indicators, such as the neonatal mortality rate, post-neonatal mortality rate, infant mortality rate, child mortality rate, and under-5 mortality rate, from the DHS data. The package allows for national and subnational indicators. In addition, the package calculates sampling error indicators such as standard error, design effect, relative standard error, and confidence interval for each demographic indicator. The package can also be used to calculate the same indicators from other population surveys such as the Multiple Indicator Cluster Survey (MICS).

## Introduction

For the last 35 years, the Demographic and Health Surveys (DHS) Program has conducted more than 300 national surveys in more than 90 countries in Africa, Asia, and South America. The DHS surveys are based on nationally representative samples that allow for national and regional estimates. Standard DHS surveys are designed to provide information about fertility, family planning, maternal and child health, and childhood mortality levels. Most of the DHS surveys follow a two-stage sampling design, where census Enumeration Areas (EAs) are selected in the first stage as Primary Sampling Units (PSUs). In the second stage, a sample of households is selected from each selected PSU. From the selected households, all women age 15–49 who slept in the household the night before the survey are eligible to complete a questionnaire designed for women. In addition, in all households or in a subsample, all men of reproductive age (typically 15–49, 15–54, or 15–59) might be eligible to complete a questionnaire designed for men.

DHS surveys can be used in many ways. In addition to the data tables published in the DHS full reports and the key indicators reports, data users can download the survey datasets in many formats, such as SPSS, SAS, and Stata, and analyze the raw data. To meet the needs of data users, the DHS releases several types of datasets for different target populations, including datasets for households, women, men, couples, children, and births. In addition to analyzing the raw data, DHS data users can use the STATcompiler to compare results across countries and trends over time, and to create customized tables and graphs [1].

Among many other questions asked of women in DHS surveys are questions about their birth history. The data on birth history and age of woman at the time of the survey are used to calculate key fertility indicators produced by the DHS surveys, such as the total fertility rate (TFR), general fertility rate (GFR), and age-specific fertility rates (ASFR). Moreover, the birth history data—especially date of birth and survival status of each live birth, and age at death of each deceased live birth—are used to calculate the following childhood mortality indicators: the neonatal mortality rate (NNMR), post-neonatal mortality rate (PNMR), infant mortality rate (IMR), child mortality rate (CMR), and under-5 mortality rate (U5MR).

Measuring indicators of fertility and childhood mortality based on household surveys such as the DHS surveys and Multiple Indicator Cluster surveys (MICS) is crucial especially in low- and middle-income countries where functional vital registration (VR) systems do not exist. Such indicators are essential to assess, either directly or indirectly, progress in meeting the United Nations (UN) Sustainable Development Goals (SDGs), especially the third goal—“Ensure healthy lives and promote well-being for all at all ages”—that calls for improving reproductive, maternal, newborn, and child health [2, 3].

Although these indicators are well defined in the literature, using survey data to calculate them has never been easy for data users. To produce these indicators, and all DHS tables, the DHS Program uses the Census and Survey Processing System (CSPro), a data processing software developed by the U.S. Census Bureau, ICF Macro, and Serpro S.A. Although CSPro is capable enough to calculate all the indicators produced by the DHS surveys, using CSPro as a statistical package has never been an option for data users. Due to the complicated calculation technique of the fertility and childhood mortality rates, users need to write complex code to replicate the rates produced by the DHS Program to use such common statistical packages as SAS, Stata, SPSS, or R. For Stata users, such code was made available through the tfr2, a Stata program to calculate fertility rates, and the SYNCMRATES, a Stata module to compute child mortality rates [4, 5]. Unfortunately for users who do not use Stata, such codes are not available yet.

Developed as an implementation of the “S” programming language, the free software environment for statistical computing and graphics “R” have been extensively used among data analysts during the last two decades. In addition to the basic functions in R, developers are able to develop and submit their own packages and the relevant documentation to the Comprehensive R Archive Network (CRAN), where data users can download the packages and use them in their analysis. These packages have extended R’s capability to accommodate specialized statistical techniques, graphical presentation, import/export capabilities, data processing capabilities, and reporting tools [6].

The main objective of this paper is to introduce the DHS.rates R package that was developed to calculate demographic indicators based on the DHS datasets, such as fertility and childhood mortality indicators [7]. In addition, the package calculates sampling error indicators, such as standard error, design effect, relative standard error, and confidence interval, for each indicator. The same package can be used to calculate indicators from other surveys, such as the MICS surveys. This paper aims to: 1) introduce readers to the DHS.rates R package; 2) demonstrate how the package can be used to calculate fertility and childhood mortality rates and their sampling error indicators based on the DHS surveys; and 3) explain how the package can be used with other surveys. To meet the last two objectives, we will illustrate how the package functions work, using several examples based on datasets from DHS and MICS surveys.

## Fertility and childhood mortality indicators

The direct estimation methods are broadly used to estimate fertility and childhood mortality indicators based on household survey data. These methods were adopted by the World Fertility Survey (WFS), which ran from 1972 to 1984, and subsequently by the DHS Program. The WFS/DHS approach is well documented in several publications [8, 9, 10, 11, 12, 13]. The WFS/DHS approach has also been used by other household survey programs, such as the MICS. In the direct estimation methods, data about the retrospective birth history are collected and used for the computation of fertility and mortality indicators [14].

In all DHS surveys, in addition to the background information collected from women age 15–49, information about birth history is collected from each interviewed woman. The birth history includes month and year of birth of each child, sex of each child, survival status of each child, age of each surviving child, and age at death of each deceased child. Such information allows for fertility rates and childhood mortality rates to be calculated from the DHS survey data. Since the rates are calculated based on birth histories collected in a survey, all rates are calculated as occurrences/exposure rates, where the numerator is the number of occurrences of some event of interest and the denominator is the population exposed to risk of the event within a reference period. In this section we will briefly introduce the definition of these fertility and childhood mortality rates and will introduce the survey variables required for calculating these rates. These definitions and methods are in line with those of the DHS, as detailed in the Guide to DHS Statistics [8].

### Fertility indicators

#### Age-specific fertility rates (ASFR)

The ASFR is the number of births occurring during a given year or a reference period per 1,000 women-years of exposure for a specific age group, in single years or five-year age groups. In the DHS surveys, ASFR is calculated as the number of births during a reference period of three years preceding the survey divided by the women-years of exposure to childbearing. It is calculated for seven age groups of five years each (15–19, 20–24, 25–29, 30–34, 35–39, 40–44, and 45–49). For each age group, where *B*_*a*_ denote the number of births to women in age group *a* during the reference period, and *E*_*a*_ denote the number of women-years of exposure in age group *a* during the same reference period, the ASFR in age group *a* can be written as follows:

$$ASFR_{a}=\left(B_{a}/E_{a}\right)\times 1000$$ (1)

From the DHS data, the information on the exact date of birth of each child can be used to directly calculate the numerator *B*_*a*_. To calculate the denominator *E*_*a*_, the exact date of birth of each woman can be used to count the number of women-years of exposure in each age group, taking into consideration that a woman can contribute to more than one age group during the reference period. For illustrations and examples about the calculation of the women-years of exposure, see [5] and [9]. In addition to measuring the fertility rate in different age groups, calculating the ASFR is crucial for measuring the TFR, as explained below.

#### Total fertility rate (TFR)

The TFR is a hypothetical measure of women’s fertility. It can be defined as the number of children who would be born per woman if she were to pass through the childbearing years bearing children according to a current schedule of age-specific fertility rates and not subject to mortality. In standard DHS surveys, *ASFR*_*a*_ is calculated for a reference period of three years preceding the survey for the seven five-year age groups. Thus the TFR can be written as follows:

$$TFR=5\times \sum_{a\in A}ASFR_{a}/1000,$$ (2)

A=(15‐19,20‐24,25‐29,30‐34,35‐39,40‐44,45‐49)

#### General fertility rate (GFR)

The GFR is the average number of children currently being born to women of reproductive age, calculated as the total number of births during a reference period divided by the total number of women-years of exposure, based on all women age 15–44 during the same reference period. In standard DHS surveys, the GFR can be written as follows:

$$GFR=\frac{\sum_{a\in A}B_{a}}{\left(\sum_{a\in A}E_{a}\right)-E_{45-49}},$$ (3)

A=(15‐19,20‐24,25‐29,30‐34,35‐39,40‐44,45‐49)

### Childhood mortality indicators

A childhood mortality rate can be defined as the number of deaths per 1,000 live births during a given year or reference period. In DHS surveys, five childhood mortality rates are calculated using a synthetic cohort life table approach, in which mortality probabilities for small age segments are combined into the more common age segments [8, 14]. The five indicators are calculated based on a reference period of five years or ten years preceding the survey and can be defined as follows:

1. Neonatal mortality rate (NNMR): the probability of dying between birth and exact age 1 month;
2. Post-neonatal mortality rate (PNMR): the probability of dying between exact ages 1 month and 1 year, usually calculated as the difference between IMR and NNMR;
3. Infant mortality rate (IMR): the probability of dying between birth and exact age 1 year;
4. Child mortality rate (CMR): the probability of dying between exact ages 1 and 5 years;
5. Under-5 mortality rate (U5MR): the probability of dying between birth and exact age 5 years.

**Fig 1:**
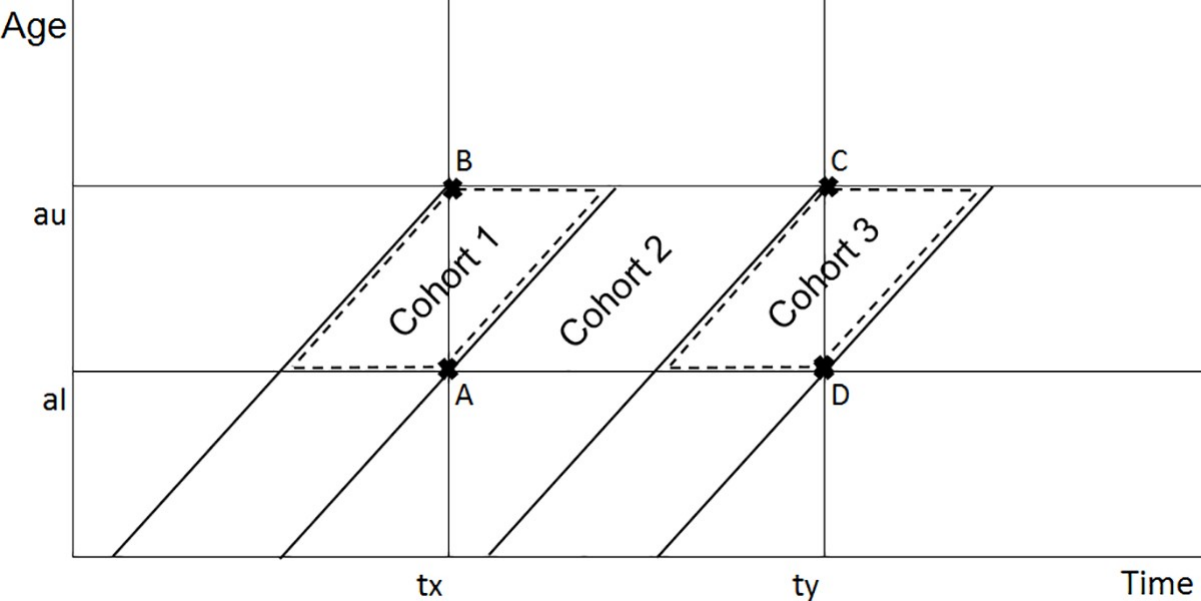
Cohorts exposed to mortality at ages a_l_ to a_u_ during the period t_x_ to t_y_.

In the DHS approach, as documented in the Guide to DHS Statistics [8], the calculations of the five mortality rates defined above start with calculating the component death probabilities for eight age segments 0, 1–2, 3–5, 6–11, 12–23, 24–35, 36–47, and 48–59 months of completed age. Each component death probability is defined by a time period and an age interval, within which three birth cohorts of children can be defined as indicated in Fig 1. As the figure shows, where the lower and upper limits of the age interval are given by a_l_ and a_u_, respectively, and the lower and upper limits of the time period are given by t_x_ and t_y_, respectively, cohort 2 (children born between dates t_x_−a_l_ and t_y_−a_u_) fully contributes to the deaths and the children-years of exposure (defined as *ABCD*), whereas cohort 1 (children born between dates t_x_−a_u_ and t_x_−a_l_) and cohort 3 (children born between dates t_y_−a_u_ and t_y_−a_l_) partially contribute to the deaths and the exposure. For example, where the reference period is the five years that ended in September 2018 (t_y_) and started in September 2013 (t_x_), for the age segment 3–5 (a_l_ = 3 and a_u_ = 6 as defined in Table 1), the three cohorts can be defined as follows:

1. Cohort 1 includes children born between March 2013 and May 2013, inclusive;
2. Cohort 2 includes children born between June 2013 and February 2018, inclusive;
3. Cohort 3 includes children born between March 2018 and June 2018, inclusive.

Note that children born between July 2018 and September 2018 are not part of the three cohorts and therefore they do not contribute to the deaths and the exposure of the component death probability for the age segment 3–5.

**Table 1:** The bounds of the age segments for the component death probabilities.

Age segment	*i*	a_l_	a_u_
0	1	0	1
1–2	2	1	3
3–5	3	3	6
6–11	4	6	12
12–23	5	12	24
24–35	6	24	36
36–47	7	36	48
48–59	8	48	60

As Fig 1 indicates, each of cohorts 1 and 3 is divided into two halves by *AB* and *CD*, respectively. Therefore, an assumption can be made that cohorts 1 and 3 are exposed to one-half of the total exposure and one-half of the deaths between ages a_l_ and a_u_ during time period t_x_ to t_y_. After the three cohorts are defined, the component death probabilities *p*_*i*_ can be calculated where *i*∈{1,2,3,4,5,6,7,8} corresponds to the eight age segments 0, 1–2, 3–5, 6–11, 12–23, 24–35, 36–47, and 48–59 months of completed age, where the age segments bounds can be defined as in Table 1.

Taking the partial and full exposure into account, the component death probabilities can be calculated as follows:

$$p_{i}=\frac{D_{i2}+0.5\times \left(D_{i1}+D_{i3}\right)}{E_{i2}+0.5\times \left(E_{i1}+E_{i3}\right)}$$ (4)

where *D*_*i1*_ denotes the number of deaths for children in age segment *i* (between ages a_l_ and a_u_) during a time period between t_x_ and t_y_, and *E*_*i1*_ denotes the number of survivors at the lower bound a_l_ of the age segment *i*, during the same time period for cohort 1. Similar definitions apply in case of *D*_*i2*_, *D*_*i3*_, *E*_*i2*,_ and *E*_*i3*_ for cohorts 2 and 3. Eq 4 is valid to calculate component death probabilities for any time period, except when the time period ends with the date of the survey. In this case, the component death probabilities should be calculated as follows:

$$p_{i}=\frac{0.5D_{i1}+D_{i2}+D_{i3}}{E_{i2}+0.5\times \left(E_{i1}+E_{i3}\right)}$$ (5)

where an assumption is made that all the deaths reported in the survey for cohort 3 for a time period that ends with the date of the survey represent one-half of the deaths that will have occurred to the cohort between ages a_l_ and a_u_.

Once the component probabilities *p*_*i*_ are calculated for each age segment, the childhood mortality rates can be calculated as follows:

$$NNMR=p_{1}\times 1000$$ (6)

$$IMR=\left(1-\prod_{i\in \left\{1,2,3,4\right\}}\left(1-p_{i}\right)\right)\times 1000$$ (7)

PNMR=IMR−NNMR (8)

$$CMR=\left(1-\prod_{i\in \left\{5,6,7,8\right\}}\left(1-p_{i}\right)\right)\times 1000$$ (9)

$$U5MR=\left(1-\prod_{i\in \left\{1,2,3,4,5,6,7,8\right\}}\left(1-p_{i}\right)\right)\times 1000$$ (10)

#### Example: The contribution of children to cohorts

**Fig 2:**
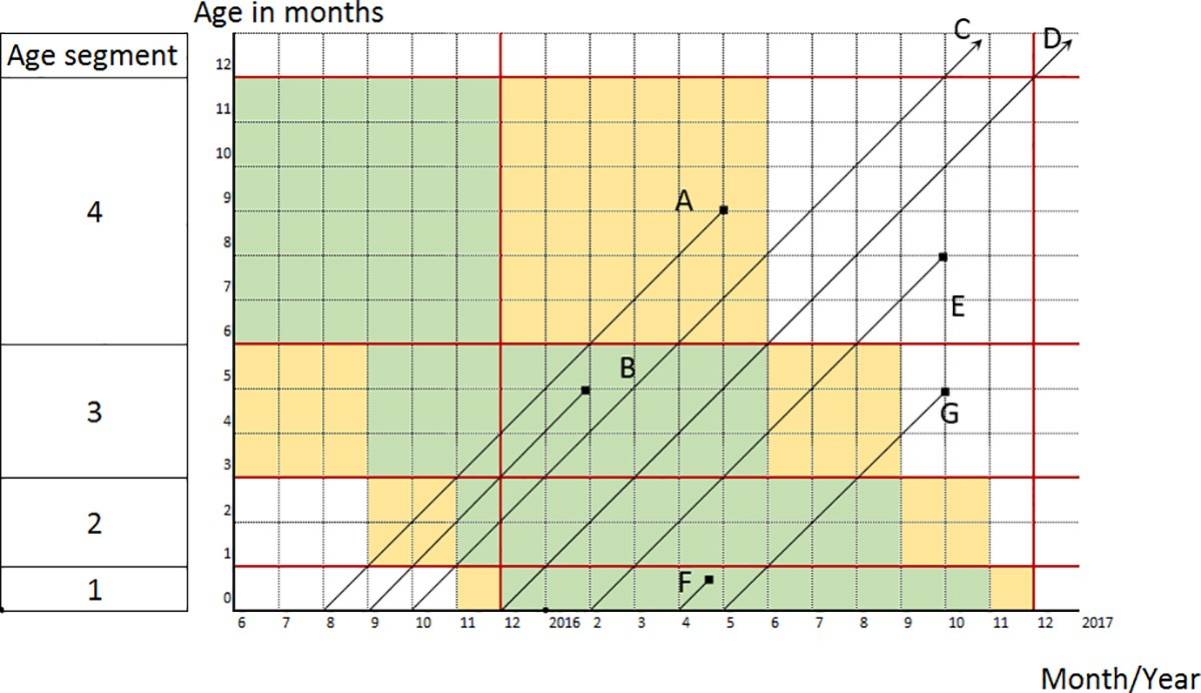
The contribution of children to cohorts.

In this example, the contribution of seven children to the different cohorts is illustrated within different age segments. To simplify the illustration, the lexis diagram in Fig 2 is limited to the first four age segments and the reference period is limited to only one year where the reference period ends on December 1^st^ of 2016, a date that precedes the date of the survey. For each of the first three age segments, the three cohorts defined in Fig 1 are indicated by colors where cohort 2 is green and cohorts 1 and 3 are yellow. For segment 4, only cohorts 2 and 3 are displayed. In Fig 2, diagonal lines are used to indicate children’s life, where deaths are indicated by dots at the end of the life line, whereas surviving children are indicated by arrows. All children were born on the first of the event month, and all children who died passed away on the first of the event month, except for child F who died during the first month of age. The seven children contribute to the component probabilities of the different age segments as follows:

1. Child A, born in August 2015 and died in May 2016, belongs to cohort 1 of age segment 3 and cohort 2 of age segment 4, and therefore this child partially contributes to the exposure of *p*_*3*_ and fully contributes to the exposure and deaths of *p*_*4*_.
2. Child B, born in September 2015 and died in February 2016, belongs to cohort 1 of age segment 2 and cohort 2 of age segments 3 and 4, and therefore this child partially contributes to the exposure of *p*_*2*_, fully contributes to the exposure and deaths of *p*_*3*_, and fully contributes to the exposure of *p*_*4*_.
3. Child C, born in October 2015, belongs to cohort 1 of age segment 2 and cohort 2 of age segments 3 and 4, and therefore this child partially contributes to the exposure of *p*_*2*_ and fully contributes to the exposure of *p*_*3*_ and *p*_*4*_.
4. Child D, born in December 2015, belongs to cohort 2 of age segments 1, 2 and 3 and cohort 3 of age segment 4, and therefore this child fully contributes to the exposure of *p*_*1*_, *p*_*2*_ and *p*_*3*_ and partially contributes to the exposure of *p*_*4*_.
5. Child E, born in February 2016 and died in October 2016, belongs to cohort 2 of age segments 1, 2 and 3 and cohort 3 of age segment 4, and therefore this child fully contributes to the exposure of *p*_*1*_, *p*_*2*_ and *p*_*3*_ and partially contributes to the exposure and deaths of *p*_*4*_.
6. Child F, born in April 2016 and died before the end of the same month, belongs to cohort 2 of age segments 1, 2 and 3 and cohort 3 of age segment 4, and therefore this child fully contributes to the exposure of *p*_*1*_, *p*_*2*_ and *p*_*3*_, partially contributes to the exposure of *p*_*4*_, and fully contributes to the deaths of *p*_*1*_.
7. Child G, born in May 2016 and died in October 2016, belongs to cohort 2 of age segments 1, 2 and 3 and cohort 3 of age segment 4, and therefore this child fully contributes to the exposure of *p*_*1*_, *p*_*2*_ and *p*_*3*_, partially contributes to the exposure of *p*_*4*_, and fully contributes to the deaths of *p*_*3*_.

### Survey variables needed to calculate fertility and childhood mortality rates

As described in the definitions above, fertility rates and childhood mortality rates are calculated based on information about number of births and deaths for each woman during a reference period. This information is collected in DHS surveys under the birth history section. In DHS surveys, the birth history data are collected from all de-facto women age 15–49, or from de-facto women age 15–49 who have ever been married, where only ever-married women are eligible for the women’s interview. The birth history variables are released in two types of datasets, the women dataset (IR) and the births dataset (BR).

In the majority of DHS surveys, a full birth history is collected from interviewed women about each live-born child to whom the woman has ever given birth. The birth history section includes variables for the date of birth (month and year) of each live birth, sex of each child, survival status of each child, age of each surviving child, and age at death of each deceased child. (For more details about the DHS birth history, see [8] and [10].) In addition to the birth history variables, date of birth of each woman and date of interview are captured in the women’s questionnaire. In DHS surveys where data is collected only from ever-married women (ever-married women surveys), “all-women factors” are calculated to adjust the ever-married samples to estimate statistics based on all women. These factors have to be accommodated in calculations where the indicator is reported for all women. All dates are coded in month and year in separate variables, which are then recoded into a Century Month Code (CMC) format that corresponds to the number of months since January 1900, calculated as follows,

CMC=(Y−1900)×12+M (11)

where *Y* denotes the year of the event and *M* denotes the month of the event.

In addition to the variables needed to calculate the rates, other variables are needed to account for the sampling design and for estimating the standard error, such as the women’s survey weight, sampling cluster, and sampling strata. All information and variables needed to calculate fertility and childhood mortality rates are listed in Table 2.

**Table 2:** Survey variables needed to calculate fertility and childhood mortality rates.

Information	Variables*	Fertility	Childhood mortality
Date of interview (CMC)	v008	√	√
Woman date of birth (CMC)	v011	√	
Child date of birth (CMC)	b3_01:b3_20 (in IR files) b3 (in BR files)	√	√
Child age at death	b7_01:b7_20 (in IR files) b7 (in BR files)		√
All-women factor**	awfactt	√	
Primary sampling unit	v021	√	√
Women individual sample weight	v005	√	√
Sample strata	v022	√	√

* Names are based on the DHS datasets

** Only for ever-married women surveys

## The DHS.rates R package

In this section we introduce the DHS.rates R package that was developed to calculate fertility and childhood mortality rates from survey data. In addition to replicating the fertility and childhood mortality rates published by the DHS Program in the survey reports or in the STATcompiler, the DHS.rates package allows data users to calculate the rates:

1. for different reference periods, more or less than three years for fertility rates or more or less than five years for childhood mortality rates;
2. based on calendar years so that the end of the reference period is not necessarily the date of the survey;
3. for different sub-populations or domains other than the ones produced by the DHS Program; and
4. based on surveys other than the DHS as long as the required variables are available.

The four attributes above provide data users with great flexibility in dealing with a large number of research problems related to fertility and childhood mortality rates based on survey data. In addition, the DHS.rates package was developed to be a user-friendly tool; while the user needs only to read the survey dataset in the R environment, all the complicated calculations of the fertility and childhood mortality rates are performed in the backend of the package away from the user.

### The package functions and parameters

The current version of the DHS.rates, version 0.7.0, includes the following three functions:

1. fert to calculate the fertility rates (TFR, GFR, and ASFR) and to estimate standard error, design effect, relative standard error, and confidence interval for each rate.
2. chmort to calculate the childhood mortality rates (NNMR, PNMR, IMR, CMR, and U5MR) and to estimate standard error, design effect, relative standard error, and confidence interval for each rate.
3. chmortp to calculate the component death probabilities for eight age segments 0, 1–2, 3–5, 6–11, 12–23, 24–35, 36–47, and 48–59 months of completed age, along with the weighted and weighted total of deaths and children-years of exposure for each age segment.

More details about parameters available in each function are provided in Table 3. In addition to the three functions, DHS.rates 0.7.0 includes three artificial DHS datasets as follows:

1. ADBR70 an artificial DHS births dataset of 2,753 births and 7 variables (v005, v008, v021, v022, v025: Type of residence urban/rural, b3, and b7)
2. AWIR70 an artificial DHS all women dataset of 3,024 women and 26 variables (v005, v008, v011, v021, v022, v025, and b3_01: b3_20)
3. EMIR70 an artificial DHS ever-married women dataset of 3,014 women and 29 variables (v005, v008, v011, v021, v022, v025, awfactt: all-women factor total, awfactu: all-women factor urban/rural, awfactr: all-women factor regional, and b3_01: b3_20)

**Table 3:** Functions, parameters, and description. DHS.rates.

Function	Parameters	Description
Fert	Data.Name	The DHS women (IR) dataset or data from other survey with the same format
	Indicator	Type of fertility rate to be calculated, “tfr”, “gfr”, or “asfr”
	JK	“Yes” to estimate Jackknife standard error for TFR
	CL	Confidence level α to calculate the confidence coefficient $Z_{\frac{\alpha }{2}}$ of the confidence intervals; default if 95
	Strata	Stratification variable if other than “v022”
	Cluster	Sample cluster variable if other than “v021”
	Weight	Survey weight variable if other than “v005”
	Date_of_interview	Date of interview (CMC) variable if other than “v008"
	Woman_DOB	Woman date of birth (CMC) variable if other than “v011”
	EverMW	“Yes” for ever-married women data
	AWFact	All-women factor variable in case of EverMW = “Yes”
	PeriodEnd	The end of the exposure period in YYYY-MM format; default is the date of the survey
	Period	The study period for fertility in months; default is 36 months (3 years)
	Class	Class variable if class level indicators are needed
chmort	Data.Name	The DHS births (BR) dataset or data from other survey with the same format
	JK	“Yes” to estimate Jackknife standard error for the five rates
	CL	Confidence level α to calculate the confidence coefficient $Z_{\frac{\alpha }{2}}$ of the confidence intervals; default if 95
	Strata	Stratification variable if other than “v022”
	Cluster	Sample cluster variable if other than “v021”
	Weight	Survey weight variable if other than “v005”
	Date_of_interview	Date of interview (CMC) variable if other than “v008”
	Date_of_birth	Child date of birth (CMC) variable if other than “b3”
	Age_at_death	Child age at death (in months) variable if other than “b7”
	PeriodEnd	The end of the exposure period in YYYY-MM format; default is the date of the survey
	Period	The study period for mortality in months; default is 60 months (5 years)
	Class	Class variable if class level indicators are needed
chmortp	Data.Name	The DHS births (BR) dataset or data from other survey with the same format
	Weight	Survey weight variable if other than “v005”
	Date_of_interview	Date of interview (CMC) variable if other than “v008”
	Date_of_birth	Child date of birth (CMC) variable if other than “b3”
	Age_at_death	Child age at death (in months) variable if other than “b7”
	PeriodEnd	The end of the exposure period in YYYY-MM format; default is the date of the survey
	Period	The study period for mortality in months; default is 60 months (5 years)
	Class	Class variable if class level indicators are needed

### The functions outputs

As described in the previous section, along with the fertility and mortality rates, the fert and the chmort functions estimate standard error (SE), relative standard error (RSE), and confidence interval (CI) for each rate. Moreover, the two functions estimate design effect (DEFT) for each rate as the ratio between the standard error using the given sample design and the standard error that would result if a simple random sample had been used. In addition the two functions provide the weighted and the unweighted women-years and children-years of exposure, (WN) and (N).

The methods of calculating the standard errors in the DHS.rates package are in line with the DHS approach detailed in the DHS Sampling and Household Listing Manual [15]. Since the ASFR and GFR can be classified as ratio estimates, where a numerator *y* can be *B*_*a*_ or ∑_*a*∈*A*_*B*_*a*_ and a denominator *x* can be *E*_*a*_ or (∑_*a*∈*A*_*E*_*a*_)−*E*_45−49_ (see Eqs 1 and 3), the Taylor linearization method is used to estimate the SE for the ratio *r* = *y/x* as follows:

$$se\left(r\right)=\sqrt{\frac{1}{x^{2}}\sum_{h=1}^{H}\left[\frac{m_{h}}{m_{h}-1}\left(\sum_{i=1}^{m_{h}}{\left(y_{hi}-rx_{hi}\right)}^{2}-\frac{{\left(y_{h}-rx_{h}\right)}^{2}}{m_{h}}\right)\right]}$$ (12)

where *m*_*h*_ denotes the total number of clusters selected from stratum *h*, *y*_*hi*_ denotes the sum of the weighted values of variable *y* (*B*_*a*_ or ∑_*a*∈*A*_*B*_*a*_) in cluster *i* in stratum *h* and *x*_*hi*_ denotes the sum of the weighted exposures (*E*_*a*_ or (∑_*a*∈*A*_*E*_*a*_)−*E*_45−49_) in cluster *i* in stratum *h*.

Unfortunately, using the Taylor linearization method is not possible in the case of complicated indicators, such as the TFR and childhood mortality rates. In this case, replication methods, such as the Jackknife repeated replication method, can be used to estimate the standard error based on replications from the survey sample. According to the DHS approach, a non-stratified Jackknife repeated replication is used, where each replication considers all but one cluster in the calculation of the estimates. Therefore, the total number of replicates should be the same as the total number of sample clusters in the survey. Where a rate *r* can be the TFR or any of the childhood mortality rates, the standard error of a rate *r* is calculated as follows:

$$se\left(r\right)=\sqrt{\frac{1}{k(k-1)}\sum_{i=1}^{k}(kr-(k-1)r_{\left(i\right)}-r{)}^{2}}$$ (13)

where *r*_*(i)*_ denotes the rate *r* calculated based on the replicate *i* where cluster *i* is excluded, and *k* denotes the total number of replicates or clusters.

The standard errors in Eqs (12) or (13) are used to calculate RSE, the ratio between *se(r)* and rate estimate *r*, and confidence interval (CI) as *r* ± $Z_{\frac{\alpha }{2}}$ *se(r)*.

### Illustrations

In this section, several examples will illustrate the use of DHS.rates functions (fert, chmort and chmortp) to calculate the fertility rates (ASFR, TFR, and GFR), the childhood mortality rates (NNMR, PNMR, IMR, CMR, and U5MR), and the childhood component death probabilities. The indicators will be calculated based on different reference periods. The examples will be based on data from the DHS and other surveys such as the MICS. Datasets from the following surveys will be used in the illustrations: the all-women 2015 Zimbabwe DHS, the ever-married-women 2014 Bangladesh DHS, and the 2017 Sierra Leone MICS. The datasets can be downloaded from the DHS and MICS webpages.

### Examples of fert

The following example shows how to calculate fertility rates (ASFR, TFR, and GFR) based on data from a DHS survey. In the code below, in addition to installing and calling the DHS.rates package, it is recommended to install and call the haven package that will allow for reading the data into the R environment [16]. In this example, the read_dta function was used to read a Stata file of the women (IR) dataset of the 2015 Zimbabwe DHS (ZDHS) that is defined as ZWIR71 in the R environment.

> install.packages("DHS.rates")
> library(DHS.rates)
> install.packages("haven")
> library(haven)
> ZWIR71 <- read_dta("C:/………………/ZWIR71FL.DTA")

In the code below, the fert function is used to calculate the ASFR, where the Indicator argument is declared as "asfr". In addition to the rates for the seven age groups, the function produces standard errors (SE), exposures (N), weighted exposures (WN), design effect (DEFT), relative standard error (RSE), and confidence interval lower bound (LCI) and upper bound (UCI). The function results commence with a description of the reference period length (36 months), the period end date (the time of the interview, in 2015.75 or Jul—Dec 2015) and the average reference period (2014.25).

> fert(ZWIR71,Indicator = "asfr")
The current function calculated ASFR based on a reference period of 36 months
The reference period ended at the time of the interview, in 2015.75 OR Jul—Dec 2015
The average reference period is 2014.25
  AGE   ASFR   SE   N   WN   DEFT   RSE   LCI   UCI
0 15–19 110.005 4.889 6071 6031 1.274 0.044 100.423 119.587
1 20–24 204.500 5.994 5114 4949 1.210 0.029 192.751 216.249
2 25–29 201.160 6.374 4960 4998 1.250 0.032 188.667 213.653
3 30–34 147.012 5.693 4506 4590 1.153 0.039 135.854 158.169
4 35–39 102.020 5.658 3465 3522 1.125 0.055 90.930 113.109
5 40–44 34.087 4.021 2509 2486 1.097 0.118 26.206 41.968
6 45–49 6.086 2.275 1123 1092 0.982 0.374 1.627 10.544

The code above is equivalent to the code below, where several arguments are explicitly identified and set to the default values in the function. Some of these arguments are very helpful, especially where datasets other than the DHS are used.

> fert(ZWIR71,Indicator = "asfr", CL = 95, Cluster = "v021",
+ Strata = "v022", Weight = "v005",
+ Date_of_interview = "v008",
+ Woman_DOB = "v011",
+ Period = 36)

The function results can be saved in separate matrix, ZW_asfr, as follows:

> ZW_asfr <- fert(ZWIR71,Indicator = "asfr")

Similarly, the fert function can be used to calculate the GFR or the TFR, where the Indicator argument is declared as "gfr" or "tfr" respectively, as below.

> fert(ZWIR71,Indicator = "gfr")
The current function calculated GFR based on a reference period of 36 months
The reference period ended at the time of the interview, in 2015.75 OR Jul—Dec 2015
The average reference period is 2014.25
  GFR   SE   N   WN DEFT   RSE   LCI   UCI
[1,] 143.226 2.81 26625 26577 1.39 0.02 137.717 148.734
> fert(ZWIR71,Indicator = "tfr")
The current function calculated TFR based on a reference period of 36 months
The reference period ended at the time of the interview, in 2015.75 OR Jul—Dec 2015
The average reference period is 2014.25
    TFR     N     WN
[1,] 4.024 27748 27669

Unlike the ASFR and GFR, to produce the standard error and the other precision indicators for the TFR, the JK argument should be declared as JK = "Yes" so that the Jackknife repeated replication method will be used to estimate the standard error, as below. Note that in addition to the standard output, the function produces the number of Jackknife replicates as the number of iterations reported below.

> fert(ZWIR71,Indicator = "tfr", JK = "Yes")
The current function calculated TFR based on a reference period of 36 months
The reference period ended at the time of the interview, in 2015.75 OR Jul—Dec 2015
The average reference period is 2014.25
    TFR   SE   N   WN DEFT   RSE   LCI   UCI iterations
[1,] 4.024 0.091 27748 27669 1.4 0.023 3.845 4.203 400

In the above codes, the confidence level of the confidence intervals was set to the default 95%. The CL argument can be used to change the confidence level to 90, as below.

> fert(ZWIR71,Indicator = "gfr", CL = 90)
The current function calculated GFR based on a reference period of 36 months
The reference period ended at the time of the interview, in 2015.75 OR Jul—Dec 2015
The average reference period is 2014.25
    GFR   SE     N   WN DEFT   RSE     LCI     UCI
[1,] 143.226 2.81 26625 26577 1.39 0.02 138.603 147.848

The fert function allows for class level rates by declaring the Class argument. In the code below, the TFR is calculated for each wealth quintile.

> fert(ZWIR71,Indicator = "tfr", Class = "v190")
The current function calculated TFR based on a reference period of 36 months
The reference period ended at the time of the interview, in 2015.75 OR Jul—Dec 2015
The average reference period is 2014.25
Class     TFR     N     WN
1 poorest 5.577 4104 4696
2 poorer 4.913 3951 4616
3 middle 4.454 4219 4745
4 richer 3.729 7250 6518
5 richest 2.434 8223 7093

All the fertility rates above are calculated based on a reference period of 36 months (3 years) preceding the survey. The rates can also be calculated for different time periods; in the code below the TFR is calculated for 60 months (5 years) preceding the survey by declaring the Period argument, as below.

> fert(ZWIR71,Indicator = "tfr", Period = 60)
The current function calculated TFR based on a reference period of 60 months
The reference period ended at the time of the interview, in 2015.75 OR Jul—Dec 2015
The average reference period is 2013.25
    TFR    N    WN
[1,] 4.207 44115 43958

Moreover, the rates can be calculated for any reference period by declaring the PeriodEnd argument. In the code below, the TFR is calculated for a period that ended in September 2010 by declaring the PeriodEnd argument, as below.

> fert(ZWIR71,Indicator = "tfr", PeriodEnd = "2010–09")
The current function calculated TFR based on a reference period of 36 months
The reference period ended in 2010.75 OR Sep 2010
The average reference period is 2009.25
    TFR    N    WN
[1,] 4.251 21666 21624

The package also is set to warn the user if the specified PeriodEnd extends beyond the dates of the survey. In the example below, the PeriodEnd was set to October 2016, although the survey fieldwork was in July-December 2015.

> fert(ZWIR71,Indicator = "tfr", PeriodEnd = "2016–10")
Note:
You specified a reference period that ends after the survey
fieldwork dates. . . . .
Make sure the dates in the survey are coded according to
the Gregorian calendar.
2. If the dates are coded according to the Gregorian calendar, use a proper PeriodEnd that came before the time of the survey.
3. If the dates are not coded according to the Gregorian calendar, use a PeriodEnd according to the used calendar.
The current function calculated TFR based on a reference period of 36 months
The reference period ended in 2016.83 OR Oct 2016
The average reference period is 2015.33
    TFR     N    WN
[1,] 2.554 28964 28933

All the functions above can be used in case of data for ever-married women. In the example below, the TFR is calculated based on the ever-married women 2014 Bangladesh DHS (BDHS) by declaring the EverMW = "Yes" argument.

> BDIR72 <- read_dta("C:/………/BDIR72FL.DTA")
> fert(BDIR72,Indicator = "tfr",EverMW = "Yes")
The current function calculated TFR based on a reference period of 36 months
The reference period ended at the time of the interview, in 2014.67 OR Jun—Nov 2014
The average reference period is 2013.17
    TFR    N     WN
[1,] 2.282 59143 59166

Moreover, the AWFact argument can be used to declare the relevant all-women factor variable especially with the Class argument, as in the code below where AWFact = "awfactu" is used in the case of calculating the TFR for urban/rural areas. Note that in the previous example the AWFact argument is not declared, since the default all-women factor variable is set to the national factor, awfactt.

> fert(BDIR72,Indicator = "tfr",Class = "v025",
+ EverMW = "Yes", AWFact = "awfactu")
The current function calculated TFR based on a reference period of 36 months
The reference period ended at the time of the interview, in 2014.67 OR Jun—Nov 2014
The average reference period is 2013.17
Class     TFR     N     WN
1 urban 2.021 21062 17272
2 rural 2.391 38224 41855

#### Examples of chmort and chmortp

The following example shows how to calculate childhood mortality rates and probabilities based on data from a DHS survey, where the births (BR) dataset (ZWBR71) of the 2015 Zimbabwe DHS (ZDHS) is used. In the code below, the chmort function is used to calculate the five childhood mortality rates. Similar to the fert function, the chmort function produces SE, DEFT, RSE, and CI, when JK = "Yes" is declared.

> ZWBR71 <- read_dta("C:/………………/ZWBR71FL.DTA")
> chmort(ZWBR71)
The current function calculated Childhood Mortality Rates based on a reference period of 60 months
The reference period ended at the time of the interview, in 2015.75 OR Jul—Dec 2015
The average reference period is 2013.25
    Rate  N  WN
NNMR 28.56 6146 6447
PNNMR 21.59 5960 6245
IMR 50.14 6132 6436
CMR 19.82 5298 5540
U5MR 68.97 5644 5910
> chmort(ZWBR71, JK = "Yes")
The current function calculated Childhood Mortality Rates based on a reference period of 60 months
The reference period ended at the time of the interview, in 2015.75 OR Jul—Dec 2015
The average reference period is 2013.25
    Rate  SE  N  WN DEFT  RSE    LCI    UCI iterations
NNMR 28.56 2.60 6146 6447 1.23 0.09 23.46 33.66 400
PNNMR 21.59 2.15 5960 6245 1.20 0.10 17.38 25.80 400
IMR 50.14 3.15 6132 6436 1.16 0.06 43.97 56.31 400
CMR 19.82 2.28 5298 5540 1.45 0.12 15.35 24.29 400
U5MR 68.97 4.28 5644 5910 1.59 0.06 60.58 77.36 400

Similar to the fert function, several arguments can be used in the chmort function to declare variables with names other than the standard names used in the DHS surveys, such as Strata, Cluster, Weight, Date_of_interview, Date_of_birth, and Age_at_death. Also, arguments like CL, Class, Period, and PeriodEnd can be used in the chmort function to produce subnational rates and rates based on different reference periods and with a non-default 99% confidence interval, as below.

> chmort(ZWBR71, JK = "Yes", CL = 99, Class = "v025",
+ Period = 120, PeriodEnd = "2012–08")
The current function calculated Childhood Mortality Rates based on a reference period of 120 months
The reference period ended in 2012.67 OR Aug 2012
The average reference period is 2007.67
    Class     R     SE     N     WN     DEFT     RSE     LCI     UCI     iteration
NNMR urban 28.33 5.02 3738 3265 1.82 0.18 15.41 41.26 166
PNNMR urban 23.22 3.04 3522 3076 1.22 0.13 15.41 31.04 166
IMR urban 51.56 5.04 3622 3168 1.32 0.10 38.58 64.54 166
CMR urban 16.18 3.26 2998 2623 1.56 0.20 7.77 24.58 166
U5MR urban 66.90 6.00 3266 2861 1.57 0.09 51.44 82.36 166
NNMR1 rural 31.12 2.86 6303 7280 1.31 0.09 23.75 38.48 234
PNNMR1 rural 34.59 3.26 5990 6920 1.40 0.09 26.18 42.99 234
IMR1 rural 65.70 4.18 6191 7151 1.31 0.06 54.94 76.47 234
CMR1 rural 33.49 3.34 5150 5931 1.41 0.10 24.89 42.10 234
U5MR1 rural 97.00 6.23 5602 6458 1.65 0.06 80.96 113.04 234

Similarly, the chmortp function can be used to calculate the childhood component death probabilities, as below, where arguments like Weight, Date_of_interview, Date_of_birth, Age_at_death, Class, Period and PeriodEnd can be used in the same way as in the case of the chmortp function.

> chmortp(ZWBR71)
The current function calculated the childhood component death probabilities based on a reference period of 60 months
The reference period ended at the time of the interview, in 2015.75 OR Jul—Dec 2015
The average reference period is 2013.25
    PROBABILITY     W.DEATHS     W.EXPOSURE     DEATHS     EXPOSURE
0 0.0286 184.10 6446.84 163.0 6146.5
1–2 0.0055 34.54 6253.03 30.5 5977.0
3–5 0.0060 36.99 6192.33 38.0 5929.5
6–11 0.0109 66.87 6143.56 57.5 5870.5
12–23 0.0112 67.08 5982.26 59.5 5707.0
24–35 0.0039 22.20 5720.40 21.0 5453.0
36–47 0.0023 12.29 5410.46 11.5 5172.5
48–59 0.0026 12.96 5041.02 11.0 4843.5

#### Examples for using other surveys: MICS

The following example shows how to use the DHS.rates package with other surveys such as the MICS. In this example, the read_sav function was used to read SPSS files of the women dataset (wm) and the births dataset (bh) of the 2017 Sierra Leone MICS. In the MICS, unlike the DHS, the birth history variables are not defined in the wm dataset. Therefore in the code below, the date of birth of child (BH4C) was merged from the bh file to the wm file using the child line number (BHLN) and reformatted the same way the birth history variables are formatted in the DHS women’s datasets, where a separate variable is reserved for each birth up to maximum 20 children. In addition, the survey weight wmweight is defined. The data manipulation below requires the dcast function of the reshape2 package [17].

> library(reshape2)
> SLbh06 <- read_sav("C:/………………/bh.sav")
> SLwm06 <- read_sav("C:/………………/wm.sav")
> # keep the necessary variables only
> SLwm06 <- SLwm06[c("HH1","HH2","LN","WDOI","WDOB",
+ "wmweight","HH7")]
> SLbh06 <- SLbh06[c("HH1","HH2","LN","BHLN","BH4C")]
> SLwm06$ID <- seq.int(nrow(SLwm06))
> # merge the women data to the birth data
> SLwm06c <- merge(SLwm06,SLbh06,
+ by = c("HH1","HH2","LN"),all.y = TRUE)
>
> # recode the line number variable "BHLN" to a character
> # variable that starts with "b3_"in each cell
> SLwm06c$BHLN <- ifelse(SLwm06c$BHLN < 10,
+ paste("b3_0",SLwm06c$BHLN,sep = ""),
+ paste("b3_",SLwm06c$BHLN,sep = ""))
> # merge the date of birth variables, b3_, to the women
> # dataset
> SLwm06m <- dcast(data = SLwm06c,formula = ID~BHLN,
+ value.var = "BH4C")
> SLwm06 <- merge(SLwm06,SLwm06m,by = c("ID"),all.x = TRUE)
> SLwm06$wmweight = SLwm06$wmweight*1000000

In the code below, the fert function is used to calculate the TFR, where the function arguments are used to identify the relevant names for the sampling strata, sampling clusters, survey weight, date of interview, and women’s date of birth. In addition to calculating the TFR, the function produces a warning message to notify the user that not all the birth history variables b3_01:b3_20 existed in the data and that the missing variables were generated by the function.

> fert(SLwm06, Indicator = "tfr",
+ Strata = "HH7", Cluster = "HH1", Weight = "wmweight",
+ Date_of_interview = "WDOI", Woman_DOB = "WDOB")
The current function calculated TFR based on a reference period of 36 months
The reference period ended at the time of the interview, in 2017.58 OR May—Aug 2017
The average reference period is 2016.08
        TFR         N     WN
[1,] 4.087 50025 50006
Warning message:
In fert(SLwm06, Indicator = "tfr", Strata = "HH7", Cluster = "HH1",: Birth History variables b3_01:b3_20 are not complete; the missing variables were created

Similarly in the code below, the chmort function is used to calculate the five childhood mortality rates based on the 2017 Sierra Leone MICS.

> chmort(SLbh06, Strata = "HH7", Cluster = "HH1",
+ Weight = "wmweight", Date_of_interview = "WDOI",
+ Date_of_birth = "BH4C", Age_at_death = "BH9C")
The current function calculated Childhood Mortality Rates based on a reference period of 60 months
The reference period ended at the time of the interview, in 2017.58 OR May—Aug 2017
The average reference period is 2015.08
    Rate    N    WN
NNMR 19.92 11828 11198
PNNMR 36.21 11554 10919
IMR 56.13 11747 11130
CMR 39.86 10639 10111
U5MR 93.75 11366 10827

#### The DHS.rates web-application

All the DHS.rates functions are available for R non-users through the DHS.rates Shiny, an interactive web-based application developed using the Shiny R package [18]. The DHS.rates Shiny is hosted on a Linux server that runs the Shiny Server program. The application can be found on the DHS Program website (https://rshiny.dhsprogram.com/apps/dhs.rates/). For more illustrations see S1–S3 Figs in S1 Appendix.

## Discussion and future directions

In this paper we introduced the DHS.rates R package. The package was developed to be a useful tool for calculating fertility and childhood mortality rates based on survey data. The package is user-friendly, with several arguments to enable researchers to dig into different research topics. Although the package was designed to deal with the DHS surveys, where standard formats are used across all the datasets, the package arguments also accommodate other surveys as long as they collected the necessary data. In addition to fertility and childhood mortality rates, the package estimates precision indicators, such as standard error, design effect, relative standard error, and confidence interval.

As well as introducing the DHS.rates package functions in this paper, we presented several examples to illustrate the capabilities of the package.

The package functions are written in the R environment and are downloadable from http://www.r-project.org. The first version of the DHS.rates package (version 0.1.0) was uploaded to the CRAN in March 2018. Since then, several versions have been released where minor edits were made or new functions were introduced. All the DHS.rates functions are available in full capacity for R non-users through the DHS.rates Shiny web-application. The DHS.rates package and the DHS.rates Shiny will continue to evolve as needed with upgrades to existing functions or new functions.

## Availability

The DHS.rates package can be downloaded from the package page on the CRAN website (https://CRAN.R-project.org/package=DHS.rates)

The DHS.rates Shiny can be found on the DHS Program website (https://rshiny.dhsprogram.com/apps/dhs.rates/).

The 2015 Zimbabwe DHS datasets can be downloaded from the data page (https://dhsprogram.com/data/dataset/Zimbabwe_Standard-DHS_2015.cfm?flag=0)

The 2014 Bangladesh DHS datasets can be downloaded from the data page (https://dhsprogram.com/data/dataset/Bangladesh_Standard-DHS_2014.cfm?flag=0)

The 2017 Sierra Leone MICS datasets can be downloaded from the data page (http://mics.unicef.org/surveys)

## Supporting information

S1 Fig DHS.rates Shiny. The web application is located in https://rshiny.dhsprogram.com/apps/dhs.rates/ and composed of three tabs: Introduction, fert and chmort. In the introduction tab a brief background is provided and instructions are outlined.
(TIF)

S2 Fig DHS.rates Shiny fert tab. The fert tab is a web application of the DHS.rates fert function. All fields in inputs panels can be modified and the relevant fertility rates are presented in the outputs panel.
(TIF)

S3 Fig DHS.rates Shiny chmort tab. The chmort tab is a web application of the DHS.rates chmort and chmortp functions. All fields in inputs panels can be modified and the relevant childhood mortality indicators are presented in the outputs panel.
(TIF)

S1 Appendix (DOCX)
