# Supplementary figures and images for: Calculating fertility and childhood mortality rates from survey data using the DHS.rates R package
Source: PLoS One. 2019 May 24;14(5):e0216403. doi: 10.1371/journal.pone.0216403 (PMC6534308; doi:10.1371/journal.pone.0216403)

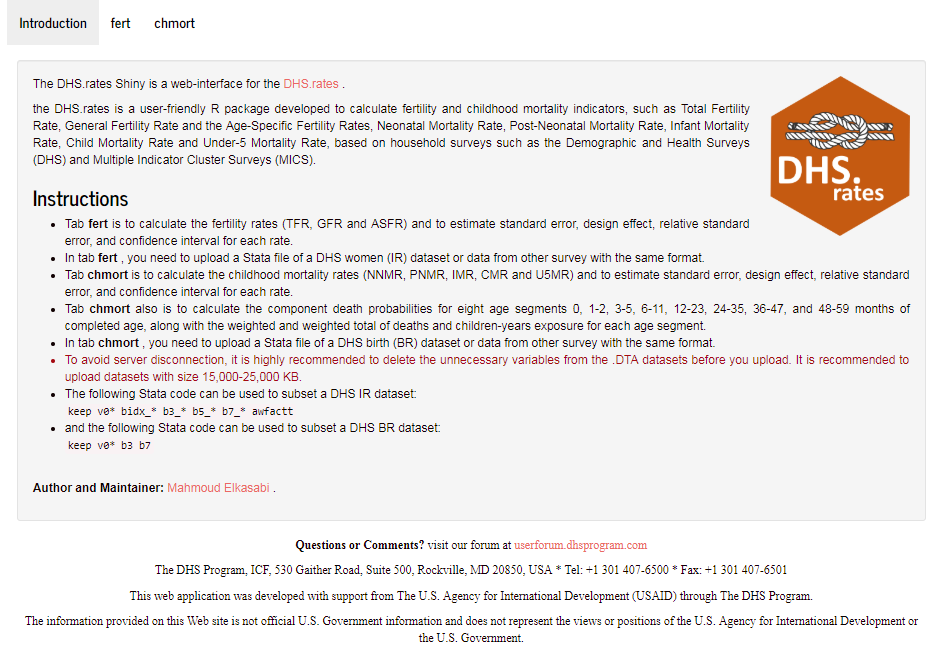

Supplement: S1 Fig — The web application is located in https://rshiny.dhsprogram.com/apps/dhs.rates/ and composed of three tabs: Introduction, fert and chmort. In the introduction tab a brief background is provided and instructions are outlined. (TIF) [file pone.0216403.s001.tif]

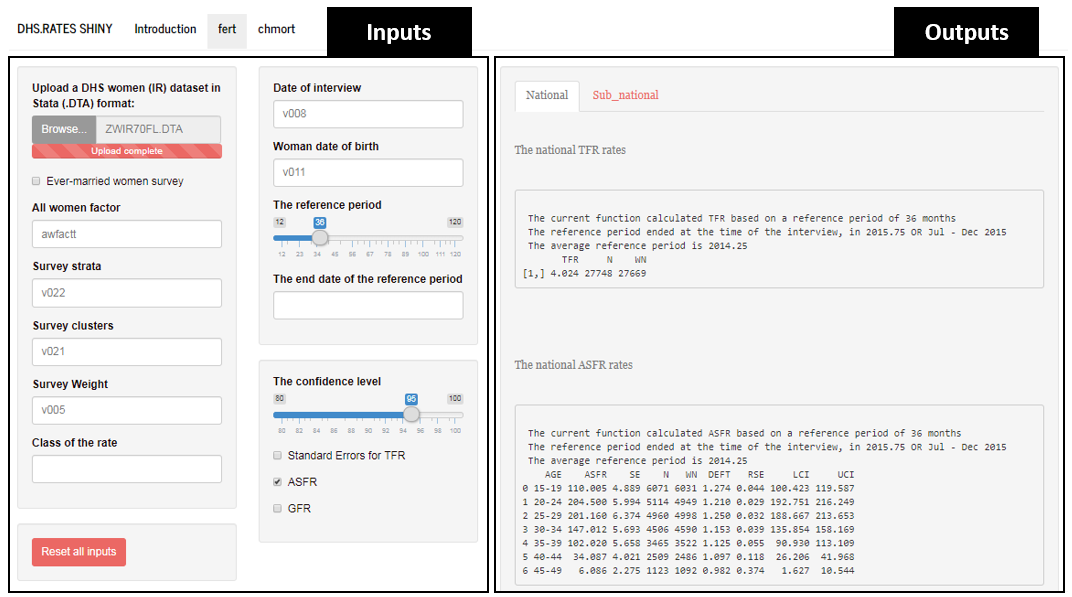

Supplement: S2 Fig — The fert tab is a web application of the DHS.rates fert function. All fields in inputs panels can be modified and the relevant fertility rates are presented in the outputs panel. (TIF) [file pone.0216403.s002.tif]

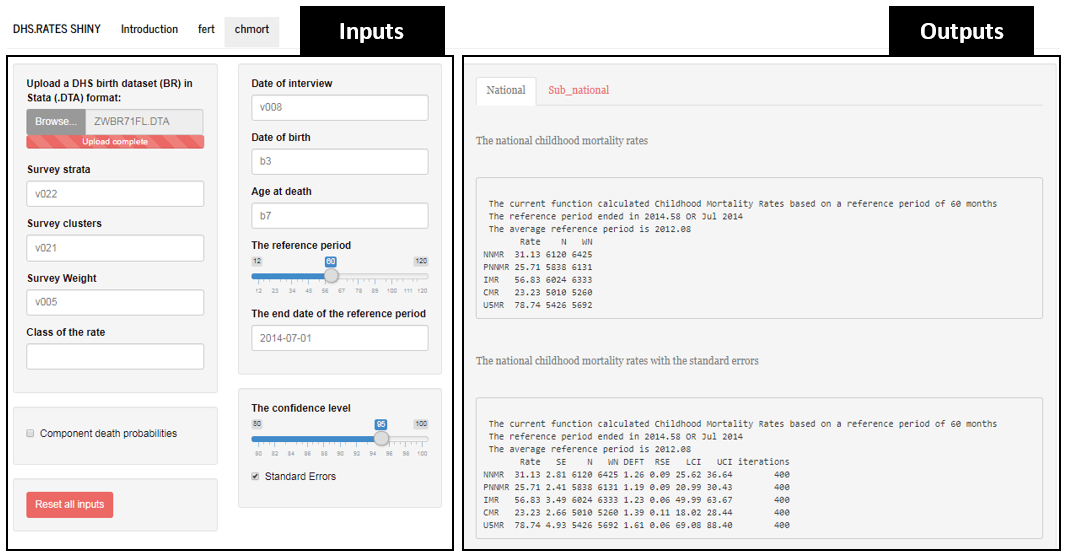

Supplement: S3 Fig — The chmort tab is a web application of the DHS.rates chmort and chmortp functions. All fields in inputs panels can be modified and the relevant childhood mortality indicators are presented in the outputs panel. (TIF) [file pone.0216403.s003.tif]
