# Supplementary material for: Calculating fertility and childhood mortality rates from survey data using the DHS.rates R package
Source: PLoS One. 2019 May 24;14(5):e0216403. doi: 10.1371/journal.pone.0216403 (PMC6534308; doi:10.1371/journal.pone.0216403)
Supplement: S1 Appendix — (DOCX) [file pone.0216403.s004.docx]

## Appendix S1: The DHS.rates Shiny application

The DHS.rates Shiny is located in <https://rshiny.dhsprogram.com/apps/dhs.rates/> and composed of three tabs: Introduction, fert and chmort. In the Introduction tab, the application is introduced and usage instructions are outlined. The fert tab is a web application of the DHS.rates fert function, where users can modify all fields in the inputs panel and get results in the outputs panel. Similarly, the chmort tab is a web application of the DHS.rates chmort and chmortp functions.

**Figure S1. DHS.rates Shiny introduction tab.** In the introduction tab a brief background is provided and instructions are outlined.

**Figure S2. DHS.rates Shiny fert tab.** In the fert tab, all fields in inputs panels can be modified and the relevant fertility rates are presented in the outputs panel.

**Figure S3. DHS.rates Shiny chmort tab.** In the chmort tab, all fields in inputs panels can be modified and the relevant childhood mortality indicators are presented in the outputs panel.
